# Using Peer Support in Developing Empowering Mental Health Services (UPSIDES): Background, Rationale and Methodology

**DOI:** 10.5334/aogh.2435

**Published:** 2019-04-05

**Authors:** Bernd Puschner, Julie Repper, Candelaria Mahlke, Rebecca Nixdorf, David Basangwa, Juliet Nakku, Grace Ryan, Dave Baillie, Donat Shamba, Mary Ramesh, Galia Moran, Max Lachmann, Jasmine Kalha, Soumitra Pathare, Annabel Müller-Stierlin, Mike Slade

**Affiliations:** 1 Department of Psychiatry and Psychotherapy II, Ulm University, DE; 2 ImROC (Implementing Recovery through Organisational Change), Department of Learning and Organisational Development, Nottinghamshire Healthcare Foundation NHS Trust, Nottingham, UK; 3 Department of Psychiatry, University Medical Centre Hamburg-Eppendorf, DE; 4 Butabika National Referral Hospital, Kampala, UG; 5 Centre for Global Mental Health, London School of Hygiene and Tropical Medicine, UK; 6 East London NHS Foundation Trust, London, UK; 7 Ifakara Health Institute, Dar es Salaam, TZ; 8 Department of Social Work, Ben Gurion University of the Negev, Beer Sheva, IL; 9 Centre for Mental Health Law and Policy, Indian Law Society, Pune, IN; 10 School of Health Sciences, Institute of Mental Health, University of Nottingham, UK

## Abstract

**Background:**

Peers are people with lived experience of mental illness. Peer support is an established intervention in which peers offer support to others with mental illness. A large proportion of people living with severe mental illness receive no care. The care gap is largest in low- and middle-income countries, with detrimental effects on individuals and societies. The global shortage of human resources for mental health is an important driver of the care gap. Peers are an under-used resource in global mental health.

**Objectives:**

To describe rationale and methodology of an international multicentre study which will scale-up peer support for people with severe mental illness in high-, middle-, and low-income countries through mixed-methods implementation research.

**Methods:**

UPSIDES is an international community of research and practice for peer support, including peer support workers, mental health researchers, and other relevant stakeholders in eight study sites across six countries in Europe, Africa, and Asia. During the first two years of UPSIDES, a series of qualitative studies and systematic reviews will explore stakeholders’ perceptions and the current state of peer support at each site. Findings will be incorporated into a conceptual framework to guide the development of a culturally appropriate peer support intervention to be piloted across all study sites. All intervention and study materials will be translated according to internationally recognised guidelines.

**Expected Impact:**

UPSIDES: will leverage the unique expertise of people with lived experience of mental illness to strengthen mental health systems in high-, middle- and low-income countries. UPSIDES will actively involve and empower service users and embed patient-centeredness, recovery orientation, human rights approaches, and community participation into services. The focus on capacity-building of peers may prove particularly valuable in low-resource settings in which shortages of human capital are most severe.

## Introduction

Peer support has been defined as “a direct service that is delivered by a person with a serious mental illness to a person with a serious mental disorder… This specialized assistance offers social support before, during, and after treatment to facilitate long-term recovery in the community in which the recovering person resides” [1, p. 430]. Peer support is part of a broader recovery agenda which places more emphasis on person-centred outcomes, such as social inclusion and empowerment, rather than traditional clinical outcomes, such as psychiatric symptomatology. Peers can support their own recovery and the recovery of others through practical and emotional support, positive self-disclosure, promoting hope, empowerment, self-efficacy, and expanding social networks [1,2,3,4]. Peers can also provide a wide range of services, including social support, disease management, counselling, outreach, coaching, and advocacy [5], which are formalised in specially-designed peer positions, such as peer companions, peer advocates, consumer case managers, peer specialists, or peer counsellors [6]. Peer support can be provided in different settings as an alternative to an independent service within or an integral part of professional care [7]. As such, it provides mechanisms for people with lived experience of mental illness to engage with those whose need for support is high, but who are often alienated from traditional health services [6].

### Evidence for peer support

Both qualitative and quantitative studies have demonstrated the far-reaching impact of peer support in high-income countries (HICs). Positive effects include improved empowerment, hope, quality of life, self-esteem, social inclusion, and engagement with care for service users [1,8,9,10]; better functioning, recovery, social networks, and employment for peer support workers (PSWs) [8,11,12,13]; and improved attitudes of staff toward service users, skills mix, recovery-orientation, and cost savings for service providers [8,14,15,16,17,18]. A systematic review identified eleven randomised trials in HICs involving 2,796 people, showing that PSWs achieved similar outcomes to professionals employed in similar roles [19]. Another systematic review based on 20 studies, including quasi-experimental trials, concluded that compared with professionals, PSWs were better at reducing inpatient service use and at improving the relationship with providers, engagement with care, and a variety of recovery-related outcomes (empowerment, behavioural activation, hopefulness for recovery) in people with severe mental illness (SMI) [14]. Overall, the evidence suggests that peer support contributes to improvements in mental health service responsiveness, safety, effectiveness, efficiency, and in making services more person-centred [3,8]. Moreover, PSWs are better than professionally qualified staff at promoting recovery outcomes such as hope, empowerment, self-esteem and self-efficacy, social inclusion, and engagement [8,19].

### Global implementation of peer support

Service users and researchers in HICs have advocated strongly for access to peer support for people with SMI [20,21,22]. In recent years, peer support has been adopted into policy in many English-speaking HICs (USA [23], Australia [24], New Zealand [25], Canada [26], UK [27]). The spread of peer support is also increasing across Europe. In German-speaking countries, there is a long tradition of “trialogue” projects which bring together people with psychosis, carers, and professionals in order to initiate role change [28]. More recently, public relations and anti-stigma projects have been introduced, including the development of new roles such as “life teacher” [29], paving the way for peer support [30]. Ten years ago, “Experienced Involvement” (peer counselling education curriculum) was initiated by an EU-funded project in six European countries (Germany, UK, Netherlands, Norway, Slovenia, and Sweden) and has since gained popularity [28]. In Israel, service users participate in decision making processes of government policy-makers. The Ministry of Health initiated and funded training and implementation of consumer-provider programs in order to integrate users as providers in mental health services [31,32].

Despite a growing evidence base on the effectiveness and cost-effectiveness of task-sharing approaches [33], in which responsibilities for mental health care are shared between mental health specialists and non-specialists such as community health workers, there is considerably less evidence on peer support from low- and middle-income countries (LMICs). Two systematic reviews have shown positive results of peer-delivered interventions, but the reviews did not define peers as people with lived experience of mental illness [2,34]. However, there are several promising examples of peer support programmes initiated in LMICs in recent years.

In Uganda, the Brain Gain projects have developed a peer support programme currently based at Butabika National Referral Hospital and serving urban and semi-urban communities in and around Kampala [35]. A case study of the first Brain Gain project demonstrated the feasibility and acceptability of peer support for people living with SMI as a strategy to drastically increase contact coverage of community mental health services in low-resource urban areas [36]. In India, the QualityRights Gujarat project funded by Grand Challenges Canada has also trained PSWs as part of a broader package of mental health system reform aimed at improving compliance with the United National Convention on the Rights of Persons with Disabilities [37].

### Rationale and objectives

Although there is a growing body of research on peer support, common definitions of peer support service types, values, standards, models, manuals, training curricula, and fidelity measures are lacking [1,14]. More evidence is needed on the central features of peer support, such as the setting and mode in which it is delivered, as well as the background and responsibilities of peers, in order to better tackle challenges, such as ill-defined roles and resistance among staff [38,39,40]. Further, cultural competence should be addressed by evaluating the impact of ethnicity, gender, and other psychosocial or socioeconomic factors on the effectiveness of peer support [14,41]. Implementation guidelines addressing these issues have been published in HICs [27]. National approaches to defining, implementing, and evaluating peer support have also been standardized and documented [8,27]. The next stage is to develop cross-cultural and empirically-validated peer support service definitions, manuals, and fidelity measures, and to determine the outcomes which best capture the impact of peer support, for application in high-, middle- and low-income countries.

UPSIDES (Using Peer Support In Developing Empowering Mental Health Services, www.upsides.org) is a five-year, six-country study which sets out to replicate and scale-up peer support interventions for people with SMI, generating evidence of sustainable best practice in high-, middle- and low-income countries. This paper describes methods to achieve the following objectives during the first phase of UPSIDES:

1. To conduct a situational analysis of existing peer support initiatives in the participating countries in order to understand the current stage of development of peer support and identify organisational and cultural considerations of the peer support worker role which may impact development.
2. To develop a culturally appropriate peer support intervention in order to scale up peer support models where pilot initiatives already exist, and to contextualize and adapt peer support models for those sites where there are no peer support initiatives.
3. To translate and cross-culturally validate all study materials in order to ensure consistent analysis across study sites.

The peer support intervention will later be implemented and evaluated over a 36-month period in the second phase of UPSIDES, to be described in a future protocol.

## Methods

**Figure 1:**
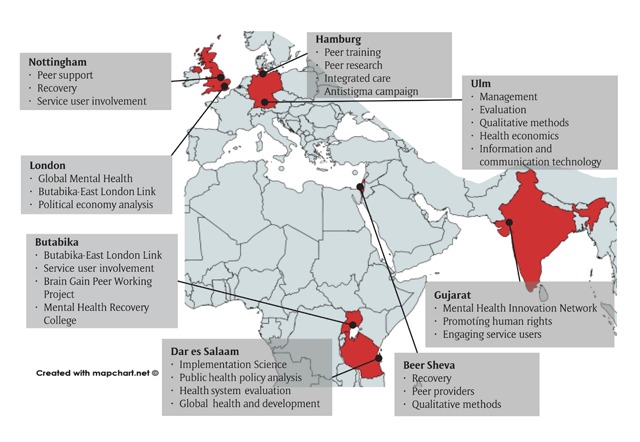
UPSIDES partners’ expertise and contributions.

UPSIDES is a collaboration of researchers at eight study sites in six countries: Ulm University, Germany; University of Nottingham, UK; Implementing Recovery through Organisational Change (ImROC), UK; University Hospital Hamburg-Eppendorf, Germany; Butabika National Referral Hospital, Uganda; London School of Hygiene and Tropical Medicine, UK; Ifakara Health Institute, Dar es Salaam, Tanzania; Ben-Gurion University of the Negev, Beer Sheva, Israel; and Centre for Mental Health Law and Policy, Pune, India. Primary data will be collected at six sites (Ulm, Hamburg, Butabika, Dar es Salaam, Beer Sheva, and Pune). Study sites represent a mix of high-income (Germany, UK, Israel), lower-middle- (India) and low-income (Uganda, Tanzania) settings and regional diversity (Europe, Eastern Mediterranean, sub-Saharan Africa, and South Asia). Figure 1 provides more information on UPSIDES partners.

**Figure 2:**
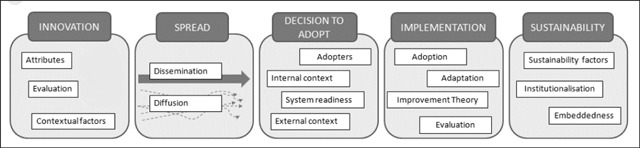
Spread and sustainability framework [42].

UPSIDES uses an implementation research framework which differentiates between five stages of innovation implementation: Innovation, Spread, Decision to Adopt, Implementation, and Sustainability (Figure 2) [42].

Study sites have been purposively selected to represent a range of different stages of scale-up, from first-time adopters (e.g. Tanzania) to those with substantial implementation experience (e.g. Uganda [36] and Israel [32]). A central principle in UPSIDES is that the PSW role is distinct and independent, rather than simply being allied to existing roles in mental health services. Independence means that PSWs services are not dependent on the service although they may be working in the service. If PSWs are linked to and paid by an outside body, it can make it easier for them to work in the interests of the person they are supporting. Level of integration will be aligned with demands for sustainability, which might require at least partial integration of peer support into existing services.

### Participants

Only participants who provide valid written informed consent will be included. Each potential participant in this research project, prior to consent, will be clearly informed of the study goals, possible adverse events, and the right to refuse to participate or to withdraw consent without any adverse consequences. Informed consent will be asked only of persons able to freely understand and question. Participants will be included if they have sufficient command of the host country’s language and are capable of giving informed consent. The study protocol has been approved by the ethics committees of all UPSIDES study sites.

#### Service users

UPSIDES addresses health needs of adults (18+) with SMI, defined as a long-standing diagnosable mental illness which has resulted in substantial functional impairment limiting major life activities. Diagnoses include psychoses such as schizophrenia, severe forms of depression, and bipolar disorder [43]. People with SMI represent a vulnerable population likely to experience significant disparities in physical health, access to and use of health care services, morbidity, and mortality in both high and low resource settings [44].

#### Peer support workers

PSWs shall be of adult age and should have experienced mental ill health, should be either in or have achieved recovery and ready to use these personal experiences, along with UPSIDES training and supervision, to facilitate, guide, and mentor another person’s recovery journey [45].

#### Other participants

Other participants include stakeholders in various roles vital to the implementation of peer support at their sites, including staff (psychiatrists, clinical psychologists, social workers, and nurses), managers, employers, and politicians.

### Research programme

The first phase of UPSIDES uses qualitative methods, including focus groups and interviews, to explore both processes and the experiences of existing PSWs, service users, and other stakeholders. This phase is divided into three work packages: Current Stage Assessment, Intervention Development, and Translation. The Current Stage Assessment work package will first develop a conceptual framework and measure for the implementation of peer support in diverse settings, and then apply this measure to each of the study sites. The Intervention Development work package will identify feasible key features and adaptable components of peer support interventions and training programs and develop flexible, ready-to-use peer service manuals and materials, including an internet-based peer support training platform. The translation work package will translate and validate all study materials for use at each study site. Further details of these work packages and their methods are provided below. Table 1 gives an overview of all UPSIDES phase 1 sub-studies collecting primary data.

**Table 1:** Features of UPSIDES studies.

Work Package	Study #	Design	Participants	Sample size per site

Current stage assessment	1.1	Focus group discussion	staff	1 group (5-8)
	1.2	Focus group discussion	stakeholders	1 group (5-8)

Intervention	2.1	Focus group discussion	stakeholders	2 groups
	2.2	Adaption test	peer support workers	2
	2.3	Adaption test	service users	6
	2.4	Focus group discussion	stakeholders	2 groups (8-12)

**Box 1** outlines UPSIDES’ standard procedures for the qualitative methods to be employed across the various work packages.

**Box 1: UPSIDES Standard Procedures for Qualitative Research.**

UPSIDES qualitative research will consist of focus groups and interviews, and will follow recommended procedures for data collection and analysis, adapted to a multinational study [46].

**Data Collection** Both focus groups and interviews will follow semi-structured topic guides. Each topic guide will cover approximately four to six topics addressing the main research questions. The topic guides will be translated from English to the local language.

Both focus groups and interviews will be audio-recorded. A moderator will facilitate the discussion and a note-taker will take detailed notes as a back-up in case of technical issues with the recording. Both the moderator and notetaker must be fluent in the local language. Immediately after the discussion, the note-taker and the moderator will write up their impressions (e.g. key themes, important insights, emotional tone) as field notes.

**Transcription and Translation** Audio files will be transcribed in line with established standards [47]. All personal details (e.g. participant names, names of people mentioned in discussion) will be anonymised both in transcripts and field notes. In order to ensure consistent analysis across sites, all transcripts and field notes will be translated into English by a bilingual speaker at each site. The initial English translation will be checked for comprehensibility and refined if needed before agreeing to the final English version.

**Analysis** Thematic analysis [48] will be used for qualitative data, grouping themes relevant to the objectives of the work package. A code book will be developed using the data from all sites, to be shared across sites for review, discussion, and standardization. The pre-determined code book will be used to develop nodes. Data will be managed into units of information covering broad categories with grouping of relevant emerging themes of importance. Each site will comment on the emergent themes. Quality will be improved by the use of multiple analysts to ensure a range of perspectives to inform the interpretation of the data, the use of verbatim quotes for each theme to ensure the interpretation is as close to the data as possible, and local validation to maximise cross-cultural validity of the coding framework.

### Current stage assessment Work Package

In this work package we will develop a theoretically-defensible and culturally-sensitive measure characterising the current stage of PSW implementation in diverse settings. A conceptual framework is a network or “plane” of interlinked concepts that together provide a comprehensive understanding of a phenomenon [49]. Conceptual frameworks are increasingly used to understand complex and multi-faceted phenomena (such as peer support implementation) within human and social systems, and offer an interpretative approach to social reality.

#### Development of a conceptual framework

We will collate international evidence from both academic and grey literature to perform two reviews: a systematic review to identify published modifications to PSW implementation and a rapid review to identify factors influencing PSW implementation. We will follow methods of narrative synthesis that have previously been used to develop conceptual frameworks for interventions to improve well-being in people with psychosis [50,51,52].

A product of our synthesis will be a preliminary conceptual framework describing the key features and implementation challenges of peer support, taking into account contextual and cultural variation across the study sites. The protocols for both reviews have been registered [53,54].

#### Validation of the conceptual framework

We will hold two focus groups at each study site to culturally validate the preliminary conceptual framework (**Box 1**). The topic guide for the focus groups will describe the purpose of the conceptual framework, and then encourage discussion by participants of local applicability, missing elements, and points of cultural adaptation (studies 1.1 and 1.2, see Table 1).

Inclusion criteria for study 1.1 will be multidisciplinary mental health workers in teams which either do or might employ PSWs. Inclusion criteria for study 1.2 will be local stakeholders with relevant expertise relating to implementation of peer work, including clinicians and managers who currently, previously, or in the future may employ PSWs, and people who currently, previously, or in the future may work as PSWs. Each focus group will comprise five to eight participants, and will take 60 minutes. Findings from the reviews and the focus groups will be synthesized to finalise the UPSIDES conceptual framework.

#### Situation analysis

A fidelity measure for peer support implementation will be developed by converting elements of the UPSIDES conceptual framework into items which can be numerically scored, with anchor points for each rating. It is likely to include assessment of cultural factors influencing implementation (e.g. issues relating to gender, presence of existing service user movements), enabling or hindering organisational influences (e.g. organisational culture, supervision capacity), and experience (if any) in implementing PSW role. Each recruiting site will be consulted to identify issues of linguistic or conceptual non-equivalence, making refinements to the UPSIDES fidelity measure where indicated. Finally, this measure will be applied to each local site to identify the current stage of implementation of peer support. We will use the findings to identify appropriate next steps in relation to PSW implementation in each site, such as organisational culture change initiatives, development of training and supervision capacity, or immediate introduction of PSW.

### Intervention development Work Package

The main task of this WP is to develop an evidence-based, culturally sensitive, manualized PSW intervention which will include a PSW training manual, a train-the-trainer manual, preparatory workshops for local service providers, and an e-learning resource for additional materials to complement face-to-face learning. Central elements of the UPSIDES intervention to be delivered by PSWs will be social support and befriending. Other elements will vary across sites depending on context, need and feasibility, and could include, for example, management, counselling, outreach, coaching, or advocacy. The intervention development will build on the conceptual framework, and the intervention will be adapted to the current stage of implementation at each site, through a literature review, expert panel, focus group discussions, and pilot.

#### Literature review

The intervention will be developed building on the conceptual framework developed in WP2 and adapted to the current stage of implementation at each site. A review of academic and grey literature will identify PSW training programmes across different resource settings, to identify: (i) core elements of PSW, based on the detection of generic key features of peer support feasible and relevant for all peer workers; and (ii) adaptable components of peer support, corresponding to local needs and goals of peer support for high-, middle- and low resource settings. The protocol for this review has been registered [55].

#### Expert panel

An expert panel will be convened with trainers from the experienced sites, to present the findings and rank identified core elements by importance so that the core training includes all elements deemed essential, with a clear rationale for excluding other elements.

#### Focus groups

After a generic intervention is developed, based on the results of the literature review and expert panel, focus groups (**Box 1**) will be undertaken at each study site with service users, providers and local stakeholders, employing a decision making tool [56] to identify mismatches between the generic intervention and partners’ views and experiences of peer support (Study 2.1, see Table 1). We will incorporate the input from the expert panels and the focus groups for the preliminary adaption design. Results of the focus group will be used to adapt the generic intervention.

#### Pilot

The preliminary adaption design will be piloted at all study sites. Two PSWs per site will work with three service users each for up to three months after receiving the preliminary training (Studies 2.2 and 2.3, Table 1). Afterward, two focus groups with relevant stakeholders will be held at each recruiting site to determine: (i) implementation difficulties, (ii) difficulties with program content or activities, (iii) satisfaction with peer support elements, including cultural features, and (iv) suggestions for improvements (e.g. language/terminology, nature of activities, specific gaps) (Study 2.4, Table 1). Continuous feedback from other staff members, peers and participants will be documented. If major barriers are identified, in-depth interviews (**Box 1**) will be conducted until saturation is reached, to ensure the problem is explored sufficiently. The preliminary intervention design will be revised based on results of the pilot, and the flexible, ready-to-use peer support intervention and training manuals and materials will be developed.

Finally, an online peer support training platform with an easy-to-use interface will be set up for PSWs to guide themselves through an online version of the intervention manual. The online platform will have the capability to include text, images, videos, audio, and questions aimed at facilitation of the peer support process, and will be designed to satisfy the technical requirements of each site. An iterative approach for content and platform development taking into account feedback from all partners of the consortium will be used.

### Translation Work Package

UPSIDES will generate three types of materials which have different translation requirements.

1. Intervention materials: The PSW intervention and training manual, as well as additional material for PSWs or the preparation workshop for mental health staff, will be developed in English, followed by local translation by a bilingual speaker.
2. Study materials: Topic guides will be developed in English, followed by local translation by a bilingual speaker (**Box 1**).
3. Qualitative data: Focus groups and interviews will generate a meeting record, comprising transcripts of the conversation (both audio-recorded and note-taker notes) and field notes completed by the interviewers after the meeting (**Box 1**). The meeting record will be transcribed into the local language by one of the interviewers or a translation service. The local language meeting record will then be translated into English (by an interviewer where possible). The English meeting record will be sent for review of comprehensibility to the Translation Work Package Leads, clarifications will be made where needed, and the English-language version will be finalised for use in analysis.

### Stakeholder involvement

Stakeholders are involved in every stage of the project, for example through local and international advisory boards. Local advisory boards provide a forum to discuss research outcomes and experiences at the local level, while UPSIDES’ international advisory board advises on and supports strategies for long-term implementation in and beyond the study sites, including the identification of key barriers and facilitators at the national and international levels. From inception, discussions are held with policy makers at each of the study sites, advising on the development of the intervention in line with the available resources in each country. Remuneration and other issues of sustainability will be a key issue in discussions with policy makers and local authorities. While salaried peer support may be necessary, especially in HICs, to ensure that adequate value is given to the work of PSWs, such payment may impede sustainability beyond the lifetime of the project in LMICs that cannot afford to add this cadre to their community health workforce. At the same time, this will ensure that the costs for replication and scale-up are minimised.

Service users are involved in various roles in this project, including as peer support providers and as service user researchers where possible. This will contribute to substantial role change for people with SMI, moving from subjects of research and recipients of treatment toward actively producing and disseminating research, and receiving training and being paid for the delivery of an evidence-based intervention to other service users.

## Discussion

There is a need to explore the effectiveness and feasibility of peer-delivered interventions for people with SMI in LMICs [2]. Peer support offers great promise to health systems with few resources where standard care is often of poor quality and low coverage [2,57]. There is also a lack of peer support for people with SMI in some components of mental health systems in HICs, such as acute care [58].

UPSIDES will:

1. Draw upon the knowledge of people with a lived experience of mental illness which is an untapped resource in global mental health.
2. Initiate organisational readiness to change so that lived experience of mental illness is recognised as a potentially valuable qualification for employment.
3. Bring about operational changes in recruitment processes and the development of new roles and practices in multidisciplinary teams alongside cultural changes in attitudes towards people with mental illness, language used, and relationships between professionals and those using services.
4. Enable peers to apply fundamental principles of peer support training such as recovery, reciprocity, mutuality, safety/trust, inclusion, and progression, whilst offering emotional and practical support, coaching, problem solving, recovery planning, and active listening.
5. Transform mental health care through peer support with its special focus on community participation.
6. Tackle the challenges of implementing peer support in LMIC, including often low organisation and status of people with mental illness.

The research to be carried out in the first phase of UPSIDES will set the foundation to implement and evaluate peer support across a range of high-, middle- and low-income countries in its second phase. The first phase of UPSIDES will focus on the development of an intervention protocol that differentiates between core ingredients of peer support which are common across all sites and those that differ between sites. The process of intervention development takes into consideration variations in the stage of implementation, as well as social, economic, cultural, and structural differences between study sites. Local ownership is emphasized because peer support will not be implemented the same in all settings, so a key deliverable will be the identification of modifiable and non-modifiable elements of the role of PSW. In the process, UPSIDES will actively involve and empower service users at all stages—as PSWs, researchers, and members of advisory boards. The result will be an evidence-based, culturally sensitive, and flexible intervention for the implementation of the peer support in a range of high-, middle- and low-income countries, from different world regions, and with different levels of experience in PSW.

## Conclusions

By focusing on actively involving people with lived experience of mental illness in the role of trained PSWs in the provision of care, UPSIDES will contribute to making health services more accessible, affordable, and equitable. Peers carry the potential for a distinct contribution not possible from traditional mental health professions such as psychiatry, psychology, and nursing. As they de-stigmatise mental illness, offer alternative viewpoints in understanding clients, and strengthen a person-focused (rather than a pathological-focused) discourse, their function is vital for users’ recovery as well as system change.
